# Development of G–Ag and C–Ag Nanoparticle‐Based Biosensor for Benzoic Acid Detection

**DOI:** 10.1002/open.202400418

**Published:** 2025-04-24

**Authors:** Mehmet Selcuk Erdogan, Muhammed Bekmezci, Nihal Yigit Ertas, Ramazan Bayat, Fatih Sen

**Affiliations:** ^1^ Sen Research Group Department of Biochemistry Dumlupinar University 43000 Kutahya Turkiye; ^2^ Altintas Vocational College Department of Chemistry and Chemical Processing Dumlupınar University 43000 Kutahya Turkiye; ^3^ Department of Materials Science & Engineering Faculty of Engineering Dumlupinar University 43000 Kutahya Turkiye; ^4^ Gediz Vocational College Department of Medical Laboratory Techniques Dumlupinar University 43000 Kutahya Turkiye

**Keywords:** Ag nanoparticles, benzoic acid, chemical synthesis, electrochemical sensors, green synthesis

## Abstract

In this study, an efficient electrochemical sensor for the highly sensitive detection of benzoic acid (BA) is developed using silver nanoparticles (Ag NPs) obtained by two different methods: the green synthesis method (G–Ag) and the chemical synthesis method (C–Ag). Linden flower extract is prepared and used for the biosynthesis of Ag NPs. Sodium borohydride, NaBH_4_, is used as a reducing agent in chemical synthesis. Ag NPs are characterized by the X‐ray diffraction (XRD) method, scanning electron microscopy (SEM), Fourier transform infrared (FTIR) spectroscopy, and UV‐visible spectrometry. According to the XRD results, the crystal sizes for G–Ag and C–Ag are calculated to be 24.07 and 5.91 nm, respectively. G–Ag and C–Ag NP‐modified glassy carbon electrodes (GCEs) and cyclic voltammetry (CV) and differential pulse voltammetry (DPV) are used as electrochemical methods to determine BA. The limits of detection of G–Ag and C–Ag NP‐modified GCEs are calculated as 1.67 mM limit of quantification and 10 mM, respectively. The linear ranges of GCEs modified with nanomaterials are determined as 2.40–8.01 mM for C–Ag and 4.84–14.66 mM for G–Ag. The study is significant in that the NPs obtained by the biological synthesis method showed as good activity as the particles synthesized by the chemical method.

## Graphical abstract

This study investigates an electrochemical sensor for the highly sensitive detection of benzoic acid on a glassy carbon electrode using silver nanoparticles produced by green synthesis (G–Ag) and chemical synthesis (C–Ag) methods. This study shows that the nanoparticles obtained through biological synthesis are as effective as those synthesized by the chemical method.

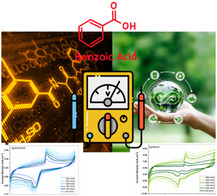

## 1 Introduction

Today, food preservation is often achieved through the use of chemical preservatives, among which benzoic and sorbic acids (BA and SA respectively), and their respective sodium, potassium, and calcium salts are commonly used.^[^ ^1^ ^]^ BA is a carboxylic acid that is widely used in the food industry as a preservative, thanks to its ability to inhibit the growth of yeast and bacteria.^[^ 2, 3 ^]^ However, more than the permitted amounts can cause metabolic acidosis, convulsions, and fungal skin diseases, such as athlete's foot and ringworm, which can be seriously harmful to the human body.^[^ ^4^ ^]^ Additionally, when reacted with ascorbic acid, benzene can be formed, which is cardiogenic, according to the World Health Organization.^[^ ^5^ ^]^ Also, adding preservatives to foods below the specified limits will not sufficiently prevent the formation of yeast, mold, and bacteria in foods. Therefore, highly sensitive BA detection is of great importance for protecting human health and quality of life. Many methods have been developed for the determination of BA in the electrophoresis,^[^ 6, 7 ^]^ spectrophotometric,^[^ 8, 9 ^]^ chromatographic^[^ ^10^ ^]^ and electrochemical^[^ 11, 12 ^]^ categories. The main disadvantages of methods other than electrochemical methods are their complexity, the cost of the devices used, and the additional skill required for the operators who will use these devices.^[^ ^13^ ^]^ In order to overcome these limitations, electrochemical analysis methods are preferred. Electrochemical sensors have become a preferred tool for the determination of biological compounds due to their extraordinary properties such as high selectivity, rapid response, low cost, and ease of operation compared to other methods.^[^ 14, 15 ^]^ The discovery of new and effective electrode materials is anticipated to improve the electrochemical detection performance of BA.

Ag‐containing nanoscale materials, Ag nanoparticles (Ag NPs), owing to their favorable chemical and physical properties, including high conductivity, excellent electrocatalytic activity, low toxicity, antibacterial properties, and remarkable stability, hold great promise for various functional devices.^[^ ^16^ ^]^ In recent years, there have been many studies on the determination of a wide variety of molecules such as glucose,^[^ ^17^ ^]^ hydrogen peroxide,^[^ ^18^ ^]^ DNA methylation,^[^ ^19^ ^]^ epirubicin,^[^ ^20^ ^]^ nifedipine,^[^ ^21^ ^]^ acyclovir,^[^ ^22^ ^]^ metronidazole,^[^ ^23^ ^]^ bisphenol‐A,^[^ ^24^ ^]^ etc. viruses^[^ ^25^ ^]^ with electrodes modified with Ag NPs.

The increasing popularity of green methods has led to the synthesis of Ag NPs using various sources such as bacteria,^[^ ^26^ ^]^ fungi,^[^ ^27^ ^]^ algae^[^ ^28^ ^]^ and plants,^[^ ^29^ ^]^ with numerous studies on this subject, resulting in less environmental pollution and large‐scale production.^[^ ^30^ ^]^ Among biological methods, the synthesis of Ag NPs using plant extracts is a more cost‐effective and environmentally friendly alternative to existing traditional chemical and physical methods.^[^ ^31^ ^]^ Another reason for choosing plants for biosynthesis is that they contain essential reducing agents, dehydrogenases, and extracellular electron carriers that may play an important role in the biosynthesis of metal NPs.^[^ ^32^ ^]^ However, molecules used in green synthesis may cause delays or deficiencies in sensor systems due to impurities.^[^ ^33^ ^]^ However, molecules used in green synthesis may cause delays or deficiencies in sensor systems due to impurities. As mentioned, in order to overcome these deficiencies, it may be necessary to turn to chemical methods. This turn has allowed the emergence of well‐characterized structures.^[^ 34, 35, 36 ^]^ BA has been determined in the current literature using various electrophoresis, spectrophotometric, chromatographic, and electrochemical techniques.^[^ ^37^ ^]^ Naturally, considering their benefits such as low cost, excellent selectivity, and fast reaction times, it is vital to develop new and more efficient electrode materials adapted to electrochemical processes.^[^ ^38^ ^]^ In this context, Ag NPs made by chemical synthesis and green synthesis (using plant extracts) have been investigated to evaluate their effectiveness for BA detection in electrochemical sensors. The benefits of the green synthesis approach over the chemical synthesis method, especially its affordability and environmental friendliness, have been an important point of emphasis.

In this study, NPs produced in two ways, green^[^ ^39^ ^]^ and chemical^[^ ^40^ ^]^ synthesis, were characterized using X‐ray diffraction (XRD) and scanning electron microscopy (SEM). For G–Ag NPs, linden flower extracts were prepared. The Ag NP‐modified glassy carbon electrode's ability to detect BA by electrochemical methods was investigated by cyclic voltammetry (CV), electrochemical impedance spectroscopy (EIS), and differential pulse voltammetry (DPV). Ag NPs have the potential to serve as an effective electrode coating material for the electrochemical sensing of BA. In addition, the green synthesized Ag NP‐based sensor is very cost‐effective, thanks to both the synthesis method and the materials used.

## 2 Results and Discussion

**Figure 1:**
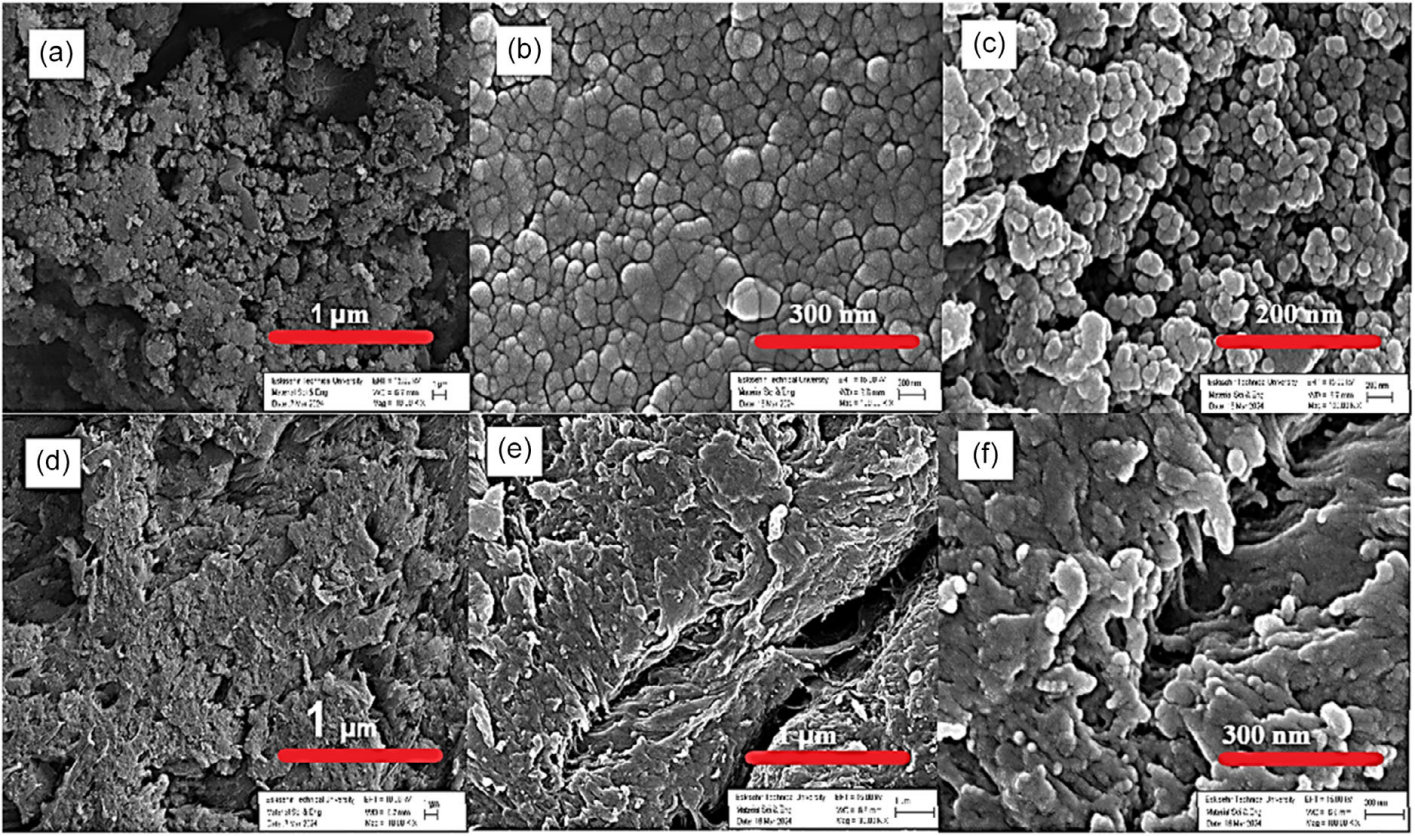
SEM images were captured at different magnifications to analyze the morphology and distribution of nanoparticles, a–c) C–Ag NPs synthesis. d–f) G–Ag NPs synthesis.

SEM, the first analysis method of the study, allowed the identification of the morphological properties of Ag NPs obtained by both synthesis methods. It was observed that the structures obtained by chemical synthesis had a relatively more homogeneous distribution than the typical Ag structure reported in the literature (**Figure** **1**a–c). Although the presence of partial agglomeration regions is noticeable in the chemical synthesis method, the presence of particles with a homogeneous distribution is generally observed. The SEM image also showed us the presence of support‐like structures originating from the plant structure. As mentioned, most importantly, in the plant‐based synthesis method, it was observed that there was a partial support structure similar to a surface area for Ag NPs to adhere to (Figure 1d–f). At the same time, almost all the particles obtained in both synthesis methods were spherical. In addition, particle distributions were relatively equal, and their distributions were quite consistent with the literature.^[^ 41, 42 ^]^

XRD analysis was also performed to determine the crystal structure of the particle, which was morphologically examined by SEM analysis. With this analysis, crystal structure and average crystal size values were obtained for G–Ag and C–Ag structures. The crystal size was calculated approximately by the Debye formula. The calculation is done with the Debye–Scherrer formula as follows (1)^[^ ^43^ ^]^

(1) $$D_{\text{hkl}}=K\lambda /\beta \cos\theta$$

where *K* is the particle shape factor, 0.9, *λ* is the X‐ray wavelength, *β*_hkl_ is the half width of reflection, and *θ* = 2*θ*/2 is the Bragg angle corresponding to the (hkl) reflection.

**Figure 2:**
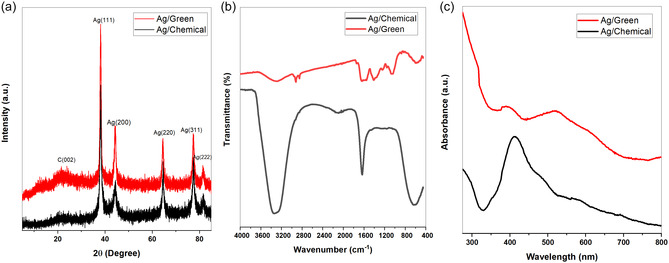
For the characterization of nanoparticles synthesized with G–Ag and C–Ag, a) XRD patterns for crystal structure analysis, b) FTIR spectroscopy patterns for the determination of functional groups, and c) UV‐VIS spectra for the examination of optical properties.

The crystal sizes calculated according to the equation are given as follows. It was confirmed by the XRD pattern of Ag NPs obtained by green synthesis and chemical synthesis, as shown in **Figure** **2**a. In both types of synthesis, Ag NPs can be noted to be in a face‐centered cubic crystal structure. The diffraction peaks appearing at the diffraction angles of ≈2*θ* = 38.16°, 44.39°, 64.44°, 77.38°, and 81.35° matched with the corresponding Miller indices (111), (200), (220), (311), and (222), respectively (JCPDS file No. 04‐0783).^[^ ^44^ ^]^ However, a peak was observed in the G–Ag structure at ≈2*θ* = 22.32°, which can be attributed to the weak and flat C (002) diffraction peak.^[^ ^45^ ^]^ It can be said that this peak is due to plant extraction and the deficiencies in washing. The average crystal size of Ag NPs in all diffraction peaks was calculated using the Debye–Scherrer formula, with an average crystal size of about 24.07 nm for G–Ag NPs and 5.91 nm for C–Ag NPs. Extra peaks appeared in the XRD pattern due to organic molecules present in the plant extract.^[^ ^46^ ^]^ FTIR was used as a further analysis to determine the bond structure between the materials. An absorption peak matching the frequency of vibration between an atom's bond in NPs is the hallmark of the particle found in its Fourier transform infrared (FTIR) resonance. The presence of functional groups inside different types of NPs may be determined using FTIR spectra since each form of NPs has a different atom configuration. Peak sizes in the spectrum can be used to estimate the amount of functional groups that are present in NPs. Using the peak value in the infrared radiation band as a guide, FTIR analysis of G–Ag NPs produced by green synthesis indicates the functional groups. C—N and C—C stretching are indicated by the major peak, which was seen at around 1622 cm^−1^.^[^ ^47^ ^]^ The stretching vibrations of the O—H groups in phenols, alcohols, and water cause the band at 3303 cm^−1^ to be seen.^[^ ^48^ ^]^ The band at 1741 cm^−1^ is caused by the acids’ carbonyl stretching vibration. The ether group and C stretching are responsible for the peak at 1048 cm^−1^.^[^ ^49^ ^]^ Additionally, signals at 589 cm^−1^ (C—Br stretching, an alkyl halide characteristic peak) and 1048 cm^−1^ (C—H stretching in alkenes) were detected. The FTIR signals of our data indicate that the soluble components in the plant extract have higher concentrations of C—N and C—C groups.^[^ 47, 50, 51 ^]^ These elements also exist as O—H group molecules, which serve as capping agents and aid in the stability of the NPs’ tract function as a sealing and stabilizing agent, which is primarily in charge of the silver ion reduction. In FTIR analysis, it is known that hydroxyl, carboxylic, phenolic, and amine groups contribute to the reduction of silver ions. The obtained FTIR spectrum confirmed this. It can be said that in the produced Ag NPs, it is mediated by aliphatic amines, aliphatic alkenes of alkaloids, and terpenoids. The band at 3359 cm^−1^ is due to the stretching vibrations of O—H groups in water, alcohols, and phenols, while the main peak observed at about 1622 cm^−1^ can be attributed to C—N and C—C stretching.^[^ ^52^ ^]^ However, in general, it presented a flat FTIR image due to metallic bonds. No significant peak value was observed in the fingerprint region for C–Ag.^[^ 53, 54 ^]^ FTIR spectra of G–Ag and C–Ag structures are given in Figure 2b.

In addition, UV‐visible spectroscopy (UV‐VIS) was also used to investigate NPs formation. Information about the size, structure, stability, and aggregation of NPs may be gathered by using UV‐VIS. UV‐VIS spectrophotometry, the best instrument for assessing metal ion reduction based on optical characteristics known as surface plasmon resonance (SPR), was used to confirm the production of Ag NPs.^[^ 55, 56 ^]^ UV‐VIS results were evaluated based on SPR properties to confirm the formation of Ag NPs. In the obtained spectrum, prominent SPR absorption bands were observed at 412 nm for the C–Ag structure and 391 nm for the G–Ag structure, indicating that the NPs were successfully synthesized. It is known that the SPR peak width can be affected by factors such as NP size distribution, shape anisotropy, and aggregation. In particular, broad absorption bands can generally indicate heterogeneous size distribution or strong interactions between NPs. The additional peak observed around 520 nm in the absorption spectrum of G–Ag NP indicates that compounds derived from herbal extracts can interact with NP and change their SPR properties. The reasons for this peak shift can be stated as polyphenols or other organic compounds in the herbal extract content binding to the NP surface and modulating the plasmonic properties. In addition, the difference in SPR peak wavelengths between C–Ag and G–Ag NPs may be due to changes in NP size and morphology. G–Ag NPs showing SPR peak at a lower wavelength (391 nm) generally indicate the presence of particles with smaller sizes and lower aggregation tendencies. UV‐VIS graphs are shown in Figure 2c.^[^ 41, 53 ^]^

**Scheme 1:**
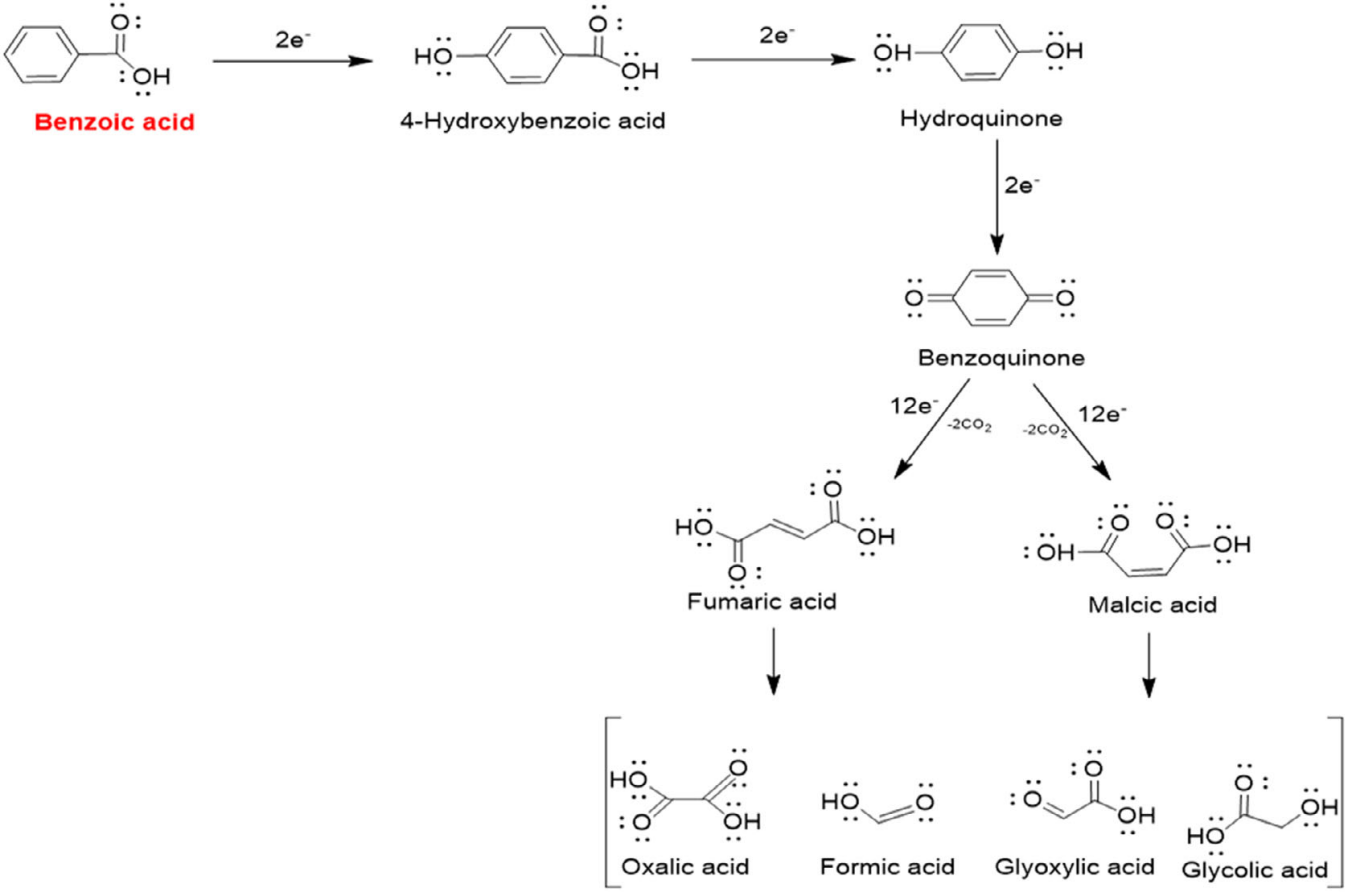
Benzoic oxidation steps (conditions: pH 6, specific to mixed sodium buffer solution components).

The results obtained by material characterization methods show that green synthesis and chemical synthesis products are very close to each other, and cleaner structures can be obtained in the chemical synthesis product. After these analysis results, the electrochemical characterization phase was started. BA has a reaction complex with different intermediate products. **Scheme** **1** shows a proposed reaction pathway for the oxidation of BA at the electrode surface based on intermediates identified in chronoamperometric experiments. These reaction steps were reported to be accessible by high erformance liquid chromatography (HPLC). The reactions show that the first oxidation step involves the formation of hydroxybenzoic acids, predominantly *para*‐hydroxybenzoic acid (4‐hydroxybenzoic acid). However, it has also been determined that there are traces of 3‐ and 2‐hydroxybenzoic acid. 4‐ Hydroxybenzoic acid passes through a sequence of consecutive reactions that result in the production of benzoquinone and hydroquinone. Additionally, phenol, catechol, and dihydroxybenzoic acids (2,4‐ and 3,4‐dihydroxybenzoic acid) were found in trace amounts. Aliphatic carboxylic acids are produced as a consequence of further oxidation processes. The accompanying data shows the HPLC peaks for maleic, fumaric, tartaric, malic, glycolic, oxalic, and formic acids, respectively. It is challenging to come to firm conclusions on the reaction process that follows ring opening because of the synthesis of many carboxylic acids at low concentrations and the inherent challenges of separating them on HPLC. However, it has been proposed that the maleic acid and fumaric acid pathway contributes to the benzoquinone oxidation process. These substances subsequently undergo further reactions to generate carboxylic acids at the C1 and C2 locations. These acids are then oxidized to CO_2_ in a process that depends on the presence of oxygen. This is followed by sequential steps to form C4 aliphatic acids, C1 and C2 carboxylic acids, and CO_2_.^[^ ^57^ ^]^

**Figure 3:**
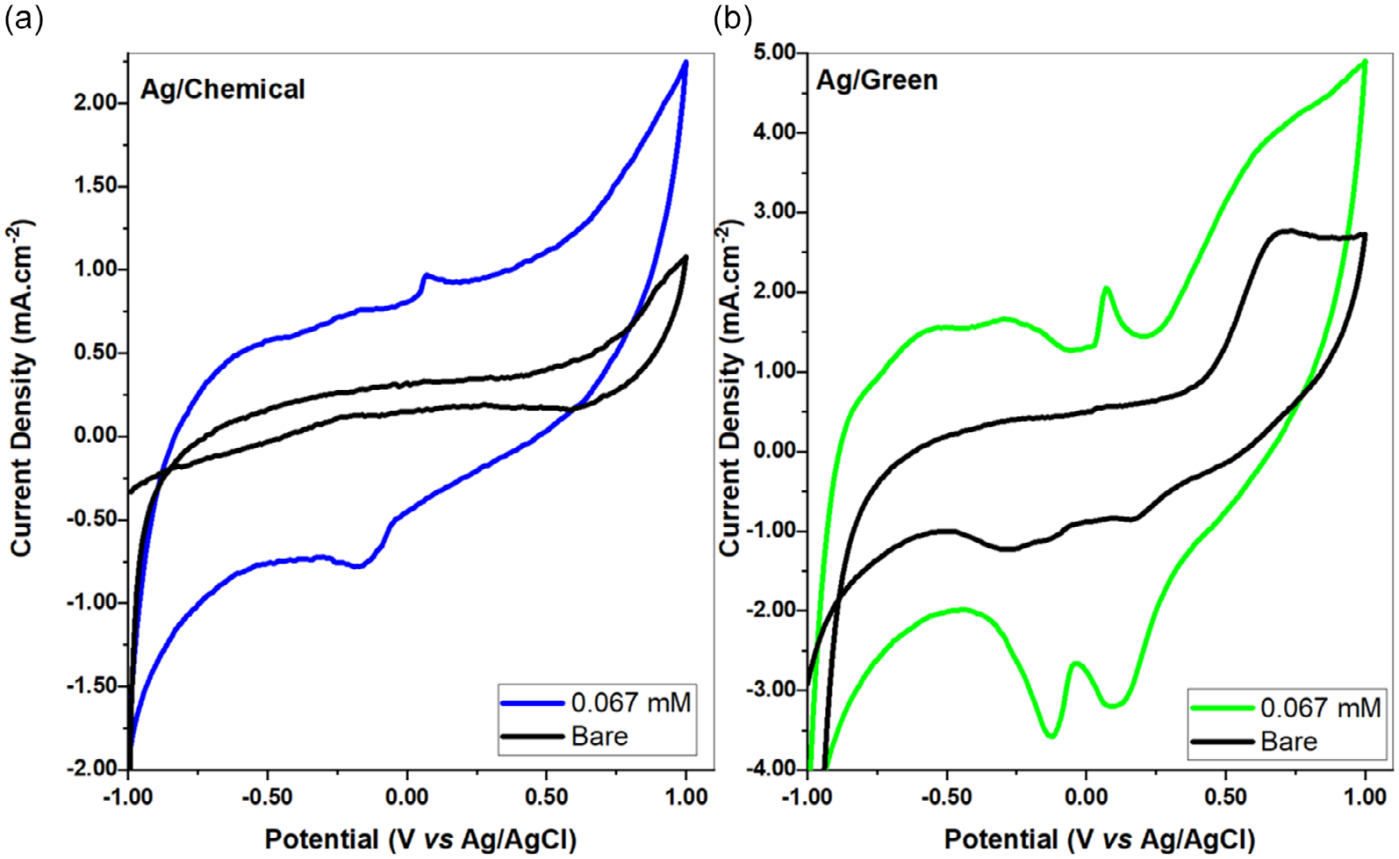
CV voltograms were calculated in 0.1 m KCl buffer solution (50 mV s^−1^ scanrate (pH:7)). a) C–Ag, b) G–Ag sensor.

G–Ag and C–Ag sensor structures were investigated based on the idea of creating a sensor depending on the oxidation degrees occurring in the reaction steps. BA sensor studies were first started with CV analysis. In all electrochemical analyses, the coating process was carried out by the drop‐casting method. Before each installation, the sensor material was sonicated for at least 15 min. Before analysis, the drying process of the electrodes was carried out in oven. The drying process was kept constant in each application and was determined as ≈30 min. Analyses were performed in a typical and fixed three‐electrode setup. The voltammetric study of BA oxidation on GCE for G–Ag and C–Ag is presented in **Figure** **3**a,b. In comparison to the blank electrode, higher current densities were seen in the voltammogram's forward scan. On the other hand, there were no pseudo‐plateaus that are supposed to indicate mass transfer restrictions, according to the literature. When compared to conventional three‐electrode configurations with fixed electrodes, they exhibit a noticeably greater mass transfer rate towards the electrode.^[^ ^57^ ^]^

DPV was used as another electrochemical analysis and calculation method. DPV analysis revealed important findings for defining the sensitivity of the sensor. In the results, very low current values were obtained in the chemical method, like the CV method, compared to the structure obtained by green synthesis. But it worked as a pretty good and effective catalyst in the nanostructure. In the structure obtained with green synthesis, the increases were seen in a partially repetitive and narrow peak line. The current appeared to be quite stable. In addition, the electrochemical resistance of the bare GCE is also important in sensor systems. This is because the ambient fluid is operated at pH 7 and changes in the environment can put stress on the sensor structure. In Figure S1, Supporting Information, the 25 cycles of bare GCE are also examined. It can be said that the catalyst produced by chemical synthesis is more stable and reaches increasing current values after 25 cycles. Although this is actually undesirable, it can be stated that this is due to the 0.1M KCl solution. In the 25 cycles with the green synthesis, it can be said that the noise is more, and the electrode gradually approaches zero current value. This may be due to the sensor structure blocking the electron pathways or being more sensitive to the ambient components.

In sensor applications, the smallest concentration of a measurand that can be accurately measured by an analytical process is referred to as sensitivity, limit of quantification (LOQ), and limit of detection (LOD). There is considerable agreement, despite the fact that several methods have been taken into consideration for these computations. The goal is to identify the lowest analyte concentration that an assay can detect without ensuring bias or imprecision of the result, the concentration at which quantitation is feasible as determined by the precision and bias targets, and, lastly, the concentration at which the analyte can be quantitatively identified with a linear response.^[^ ^58^ ^]^ Calculations in the literature have shown that the LOD and LOQ procedures are comparable to chromatographic methods. In analyses, it is defined as the sample size that give a signal‐to‐noise ratio of 3:1. Estimating the LOD using the standard deviation of the response is an alternative method to the signal‐to‐noise method. In this calculation, LOD = 3.3(SD/S), where S is the slope of the calibration curve and SD is the standard deviation of the response based on the standard deviation of the blank, the residual standard deviation of the regression line, and the standard deviation of the y‐intercept of the regression line. This method has universal validity and is a valid approach for finding the LOD. A rough estimate of the LOQ is obtained by multiplying the LOD by 3.3. The LOQ is given as a concentration‐dependent value.^[^ ^59^ ^]^

**Figure 4:**
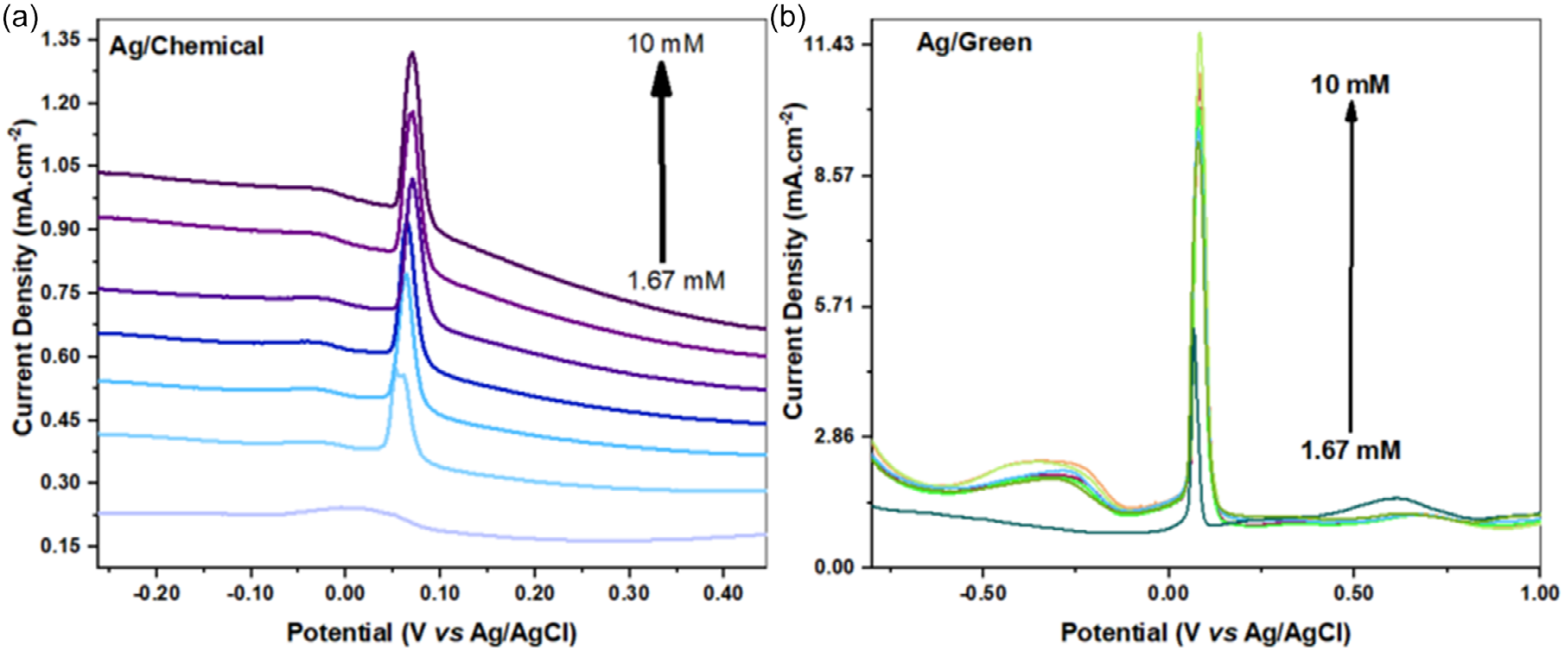
DPV voltograms were taken in 0.1 m KClsolution (pH 7). a) C–Ag sensor. b) G–Ag sensor.

To compare both sensors, the linear range was kept between 1.67 mM and 10 mM. LOD and LOQ calculations were also presented from the DPV analysis. Accordingly, LOD values for C–Ag and G–Ag were obtained as 2.40, 4.84 mM, and LOQ ratios as 8.02, 14.66 mM, respectively. The results were similar or even better than those obtained in the literature. **Figure** **4**a,b show the DPV analysis results for C–Ag and G–Ag.

**Figure 5:**
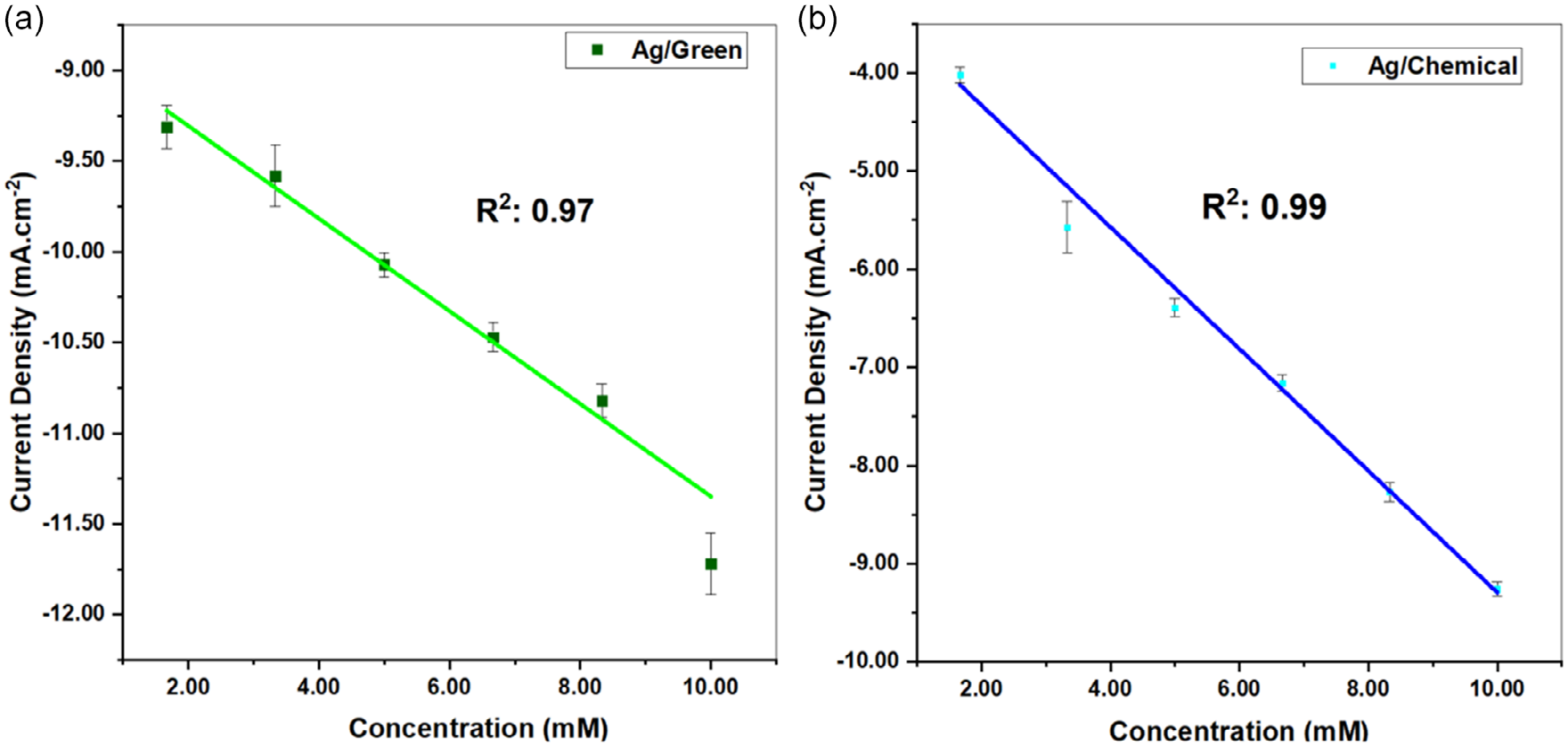
Correlation curves obtained from DPV diagrams for a) G–Ag and b) C–Ag sensors (0.1 m KCl solution, pH:7).

DPV analysis also provided us with the opportunity to establish correlations. Correlation results obtained from DPV results are given in **Figure** **5**a,b.

**Figure 6:**
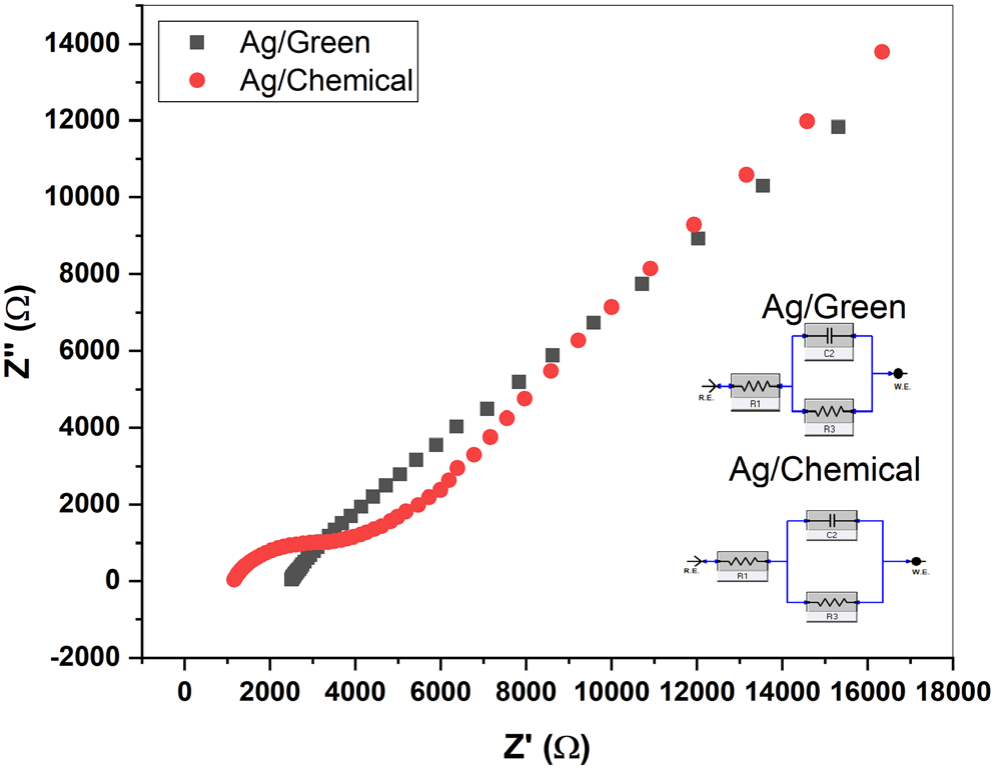
EIS (potassium ferrocyanide solution([Fe(CN)_6_]^4−^) and load circuits to study the electrical properties of C–Ag and G–Ag sensors.

Another important analysis for electrochemical analysis was performed as EIS. **Figure** **6** presents the EIS spectra obtained for G–Ag and C–Ag. It can be said that the G–Ag sensor has a slightly higher resistance than the C–Ag. This showed that faster electron transfer was achieved for the G–Ag sensor. DPV and CV analyses also confirmed this situation. C–Ag partially contained a small semicircular piece and a linear piece. Here, the semicircular section at high frequencies corresponded to the limited electron transfer process, and the linear section at lower frequencies could be said to describe the diffusion process. This situation is quite close with SEM images. Accordingly, C–Ag contains much more agglomerated structures. However, for C–Ag this situation is less significant.

**Figure 7:**
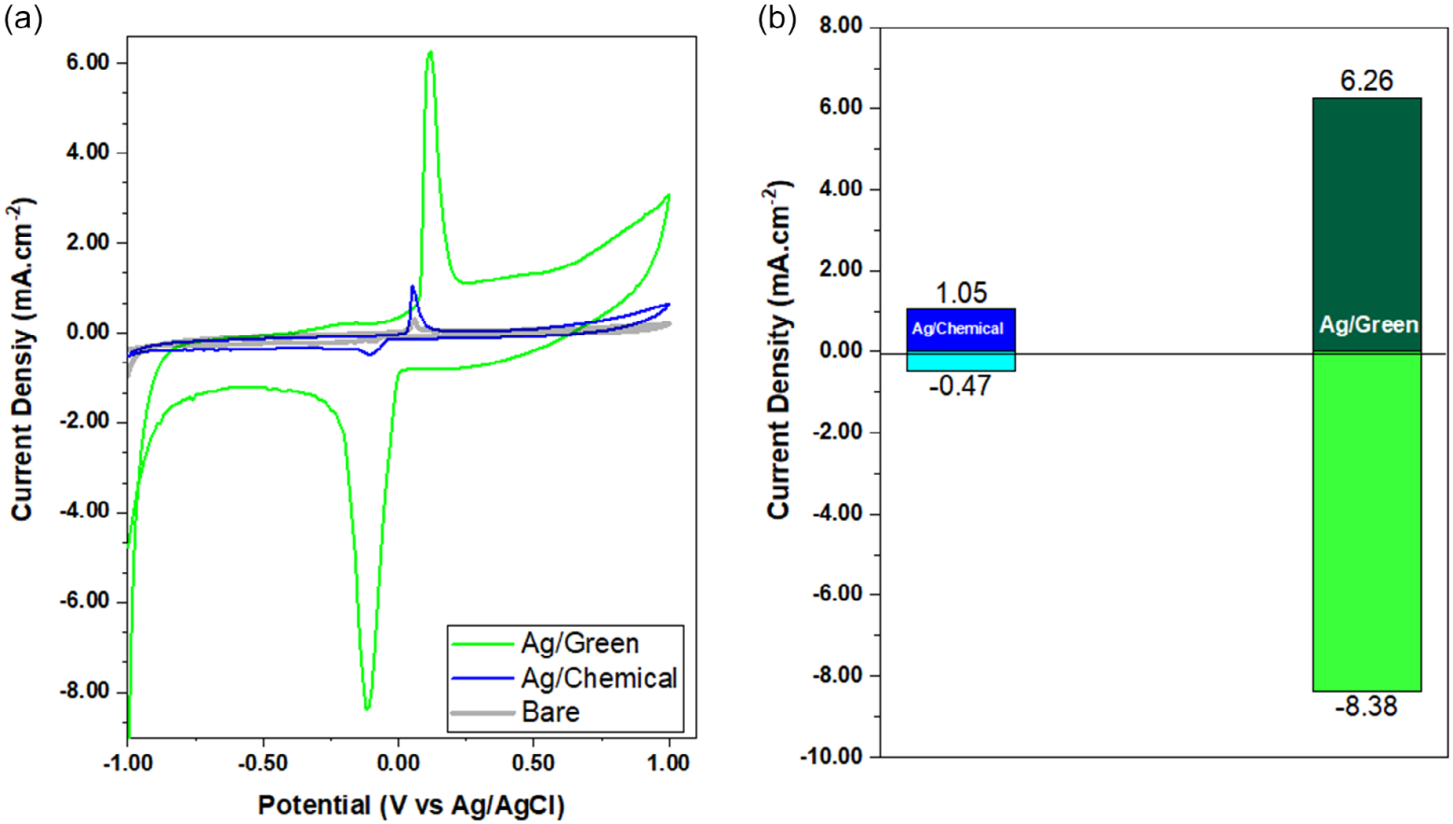
BA sensor from clove plant extract for G–Ag and C–Ag. a) CV values obtained for both sensors. b) Comparison table of forward and reverse current values calculated from CV diagrams (0.1 M KCl solution, pH:7).

Finally, according to the current values obtained, trials were carried out on the real sample. In the literature, it is seen that the materials containing the highest amount of BA are naturally found in plants and fungi.^[^ ^60^ ^]^ It is often preferred as a food additive. Rich natural sources of BA are reported to include strawberry (up to 29 mg kg^−1^), red pepper and mustard seed (up to 10 mg kg^−1^), clove, sage, thyme, and nutmeg (up to 50 mg kg^−1^), and cinnamon (up to 335 mg kg^−1^).^[^ ^61^ ^]^ Based on this, real sample measurements were taken using clove plant extract. One hundred grams of cloves were extracted in 100 mL of pure water. In the real‐time and standard three‐neck cell, 2000 μl was fed to the cell. According to the results, forward current values were obtained as 6.26 and 1.05 mA cm^−2^ for G–Ag and C–Ag, respectively (**Figure** **7**a,b). These results support the CV results, but the second step of forward reduction was not followed. According to the results, the highest current value was obtained with G–Ag, which supports the SEM and XRD results. Accordingly, taking high current values is highly related to morphology.

**Figure 8:**
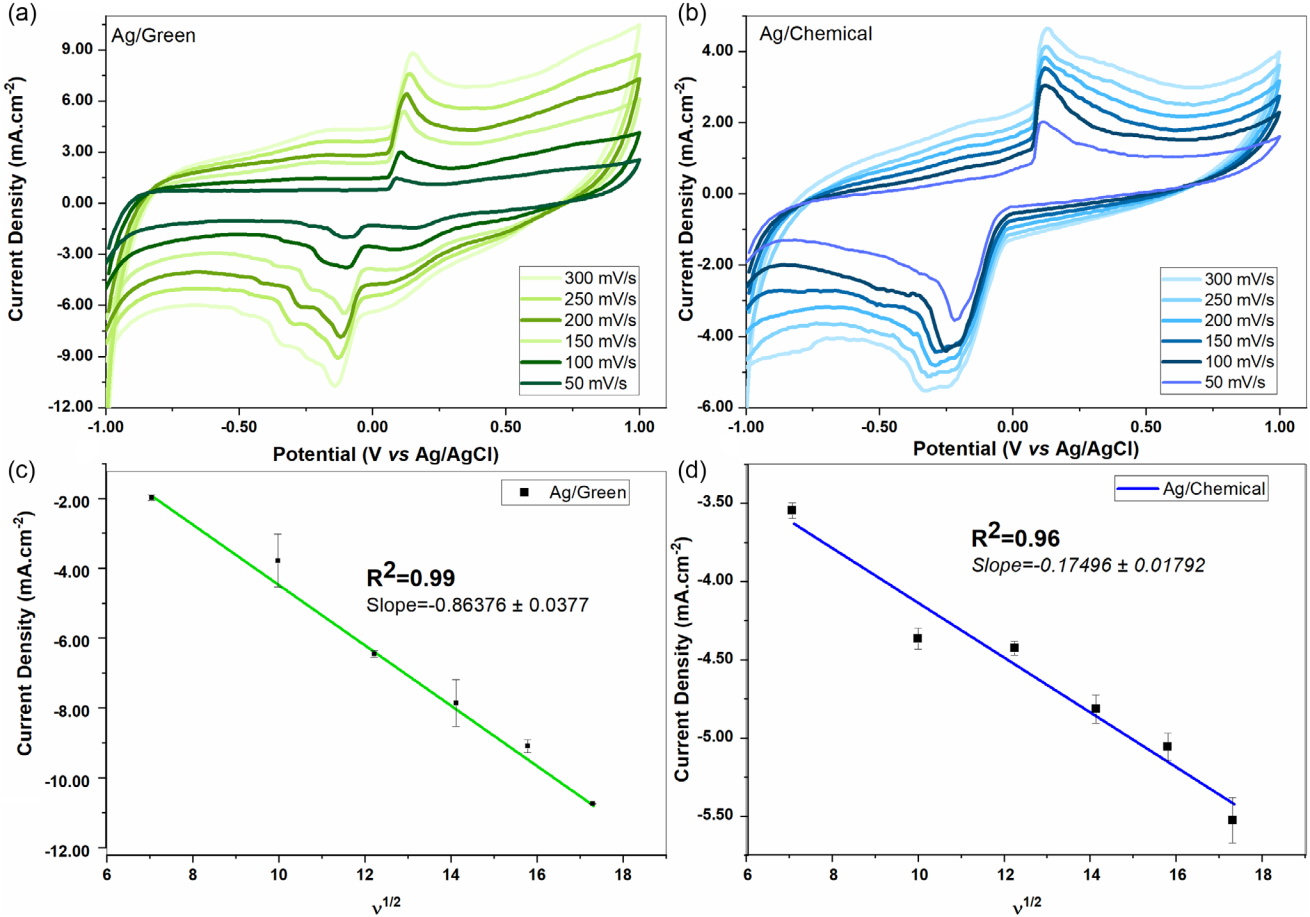
a,b) CV curves of 0.033 mM BA in GCE modified with Ag NPs synthesized by green and chemical methods in 0. 1 m KCl (pH:7) solution at different scan speeds. c,d) Linear calibration curves.

**Figure** **8** shows the CV curves in the potential range from −1 to +1 V in the modified GCE with Ag NPs using different scan rates of 0.033 mM BA in a 0.1 M KCl (pH = 7) solution. As the scan rate increased from 50 to 300 mV s^−1^, the anode peak shifted positively from − 0.5 to − 0.1 V, while the cathode peak shifted negatively from − 0.1 to − 0.19 V. The shift of anode and cathode CV peaks indicates the kinetic control of Ag NPs on the oxidation of BA. There is a good linear relationship between the anode and cathode peak currents in the scan rate range of 50–300 mV s^−1^ with υ^1/2^ . The linear regression equation given below confirms the diffusion‐controlled process in AG NPs modified GCE. However, another important issue in sensor studies is the stability of the GCE while the sensor material is in the environment. Figure S2, Supporting Information, shows the stability analysis in 0.01M BA and 0.1M KCl after 25 cycles. Although the structure produced by chemical synthesis was more stable, it exhibited typical shifts due to by‐products formed in the oxidation reactions of BA. The structure produced by green synthesis was more stable in the presence of the sensor material.

In addition, our results are compared with the data obtained in the literature in **Table** **1**. According to the results obtained, we can say that the sensor we developed makes significant contributions to the literature.

**Table 1:** BA electrochemical sensor comparison table.

Sensor	Linear detection range [mM]	LOD	Buffer solution	Electrochemical analysis	Reference
Bismuth antimonate	0.0002–2	0.053 μM	0.2 m Acetic Acid	CV	[12]
Polyaniline/CuGeO_3_ (cp_2_)	0.001–2	0.47 μM	0.1 m KCl	CV	[63]
Polythiophene/copper bismuthate nanosheet nanocomposites modified GCE	0.001–2	0.56 μM	0.1 m KCl	CV	[2]
Copper vanadate nanobelts modified GCE	0.001–2	0.61 μM	0.1 m KCl	CV	[11]
C–Ag G–Ag	1.67–10 1.67–10	2.40 mM 4.84 mM	0.1 m KCl	DPV	This Work

## 3 Conclusions

The use of BA, which is used as a food preservative, above the recommended limits causes serious diseases, and the use below the recommended limits causes food spoilage problems. To cope with this situation, it is very important to sense BA with rapid and cost‐effective methods. Based on this situation, we designed a cost‐effective electrochemical sensor using two different methods. In the analysis of the obtained structures, it was seen that the desired nanostructures were produced by both chemical and green synthesis, and both structures participated in the sensing mechanisms in a similar way. According to the XRD analysis results, the average crystal size G–Ag, and C–Ag was calculated as 24.07 and 5.91 nm from the Debye–Scherer formula. It was predicted that the differences in the crystal size were due to the reducing agent. FTIR analyses revealed the presence of both plant‐derived and chemical reduction‐derived Ag NPs components. GCEs were modified with structures obtained by both methods. The LOD of GCEs modified with G–Ag and C–Ag NPs was calculated as 4.84, 2.40, 14.66, and 8.02 mM LOQ values, respectively. The LOD of GCEs modified with G–Ag and C–Ag NPs were calculated as 4.84, 2.40, 14.66, and 8.02 mM LOQ values, respectively. A correlation graph was drawn from the calibration curves obtained, allowing the identification of BA in real samples. This study brought important results in terms of characterizing Ag NPs produced by chemical and green synthesis and comparing their performances in sensor applications. In addition, the current values obtained in the study enabled the rapid and easy detection of BA. Moreover, a very reasonable cost was achieved with the Ag NP‐based sensor synthesized using the green synthesis method.

## 4 Experimental Section

4.1

4.1.1

##### Chemicals

All purchased chemicals were of high purity and were used without any further purification procedures. Silver nitrate (AgNO_3_), sodium borohydride (NaBH_4_), nafion solution (Nafion‐117), benzoic acid (BA), potassium chloride (KCl), potassium ferrocyanide ([Fe(CN)_6_]^4−^ and N’N’‐dimethylformamide (DMF) were purchased from Sigma Aldrich. The linden tree flowers used in green synthesis were purchased from a local market.

XRD pattern of Ag NPs was obtained using Rigaku MiniFlex, SEM images were obtained from JEOL JSM 5600, and FTIR resonance graphs were obtained using FTIR/Perkin Elmer Spectrum Two. The electrodes used in the analyses whose surfaces were modified were GCE, and their glassy carbon surface diameter was 3 mm. GCEs modified with the prepared nanocatalyst were used as working electrodes, Ag/AgCl was used as a reference electrode, and a platinum plate was used as a counter electrode. GCE stability was ensured by following the following steps. Before starting the application with GCEs, the working electrodes and electrode surfaces were polished with 1.0, 0.5, and 0.3 micron alumina, respectively, and rinsed with pure water and ethanol and then left to dry at room temperature. The prepared NPs were added with 37.5 μL of dimethylformamide (DMF), 500 μL and 75 μL of Nafion solutions, respectively, and sonicated for approximately 30 minutes. 10 μL of modification solution containing Ag NPs obtained by both methods was applied to different working electrode surfaces by drop‐casting method.

##### Silver NP Green and Chemical Synthesis

Two different methods were preferred to synthesize Ag NPs: green synthesis and chemical reduction. The linden plant was preferred to be used in the production of Ag NPs via green synthesis. The linden tree flowers used in the green synthesis of Ag NPs were washed three times with pure water and left to dry in a protected environment. Typical synthesis, 1 mM AgCl was taken, and 100 mL of linden plant extract was added and reacted under constant conditions at room temperature in a 1000 mL volumetric flask. The dark solution formed by the reduction of Ag+ ions was centrifuged at 10,000 RPM for 5 min; the upper liquid phase was removed, and the remaining solid precipitate was washed several times with distilled water.^[^ ^62^ ^]^ As the second synthesis method, chemical reduction was used as mentioned.^[^ ^35^ ^]^ This method was preferred because it was a fast and relatively easy method and was briefly as follows: 1 mM AgCl was mixed in a Schlenk system at 300 RPM and argon gas atmosphere. Sodium borohydride (NaBH4), a fast‐reducing agent, was added to the medium. The Schlenk mechanism was used until gas evolution stopped. After this process, the mixed solution was centrifuged at 5000 RPM. The samples were dried in a protected atmosphere and three times with dH_2_O to eliminate unwanted chemicals in the environment, obtain a powder form, and use it for analysis. In both methods, environmental conditions were kept under control, and the synthesis aimed to produce NPs with a shape‐independent structure.

## Conflict of Interest

The authors declare no conflict of interest.

## Supporting information

Supplementary Material
