# Supplementary material for: Development of G–Ag and C–Ag Nanoparticle‐Based Biosensor for Benzoic Acid Detection
Source: ChemistryOpen. 2025 Apr 24;14(6):e202400418. doi: 10.1002/open.202400418 (PMC13062927; doi:10.1002/open.202400418)
Supplement: Supplementary file 1 — Supplementary Material [file OPEN-14-e202400418-s001.pdf]

Supplementary Materials

Development of G-Ag and C-Ag Nanoparticle-Based Biosensor for Benzoic Acid Detection

Mehmet Selcuk Erdogan[a,b], Muhammed Bekmezci[a,d], Nihal Yigit Ertas[a,c], Ramazan Bayat[a,d], Fatih Sen[a*]

[a]*Sen Research Group, Department of Biochemistry, Dumlupinar University, Kutahya, Türkiye*
[b]*Altintas Vocational College, Department of Chemistry and Chemical Processing, Dumlupınar University, Kutahya, Türkiye*
[c]*Gediz Vocational College, Department of Medical Laboratory Techniques, Dumlupinar University, Kutahya, Türkiye*
[d]*Department of Materials Science & Engineering, Faculty of Engineering, Dumlupinar University, Kutahya, Türkiye*

***Corresponding author**: fatihsen1980@gmail.com*

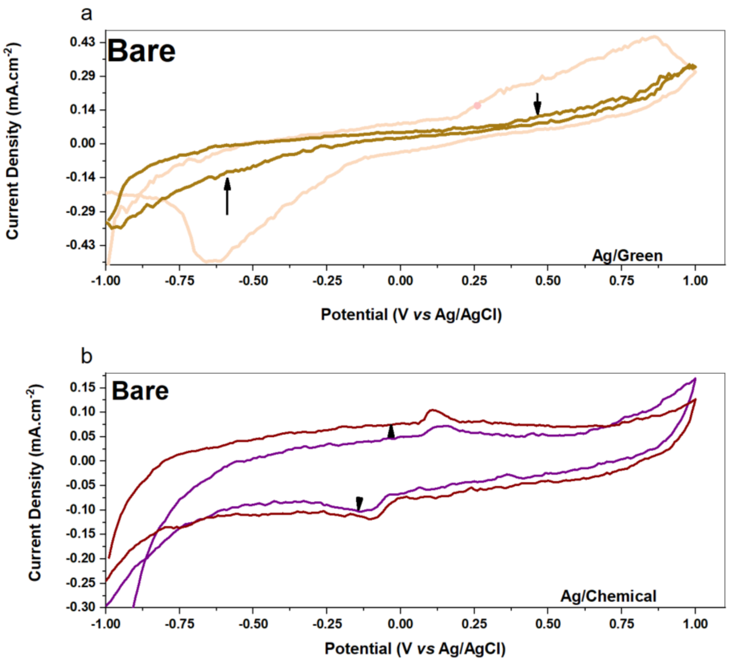

Figure S1. Stability of sensor structures after 25 cycles a) C-Ag b) G-Ag.(1 M KCl, 0 M BA and 50 mV/s)

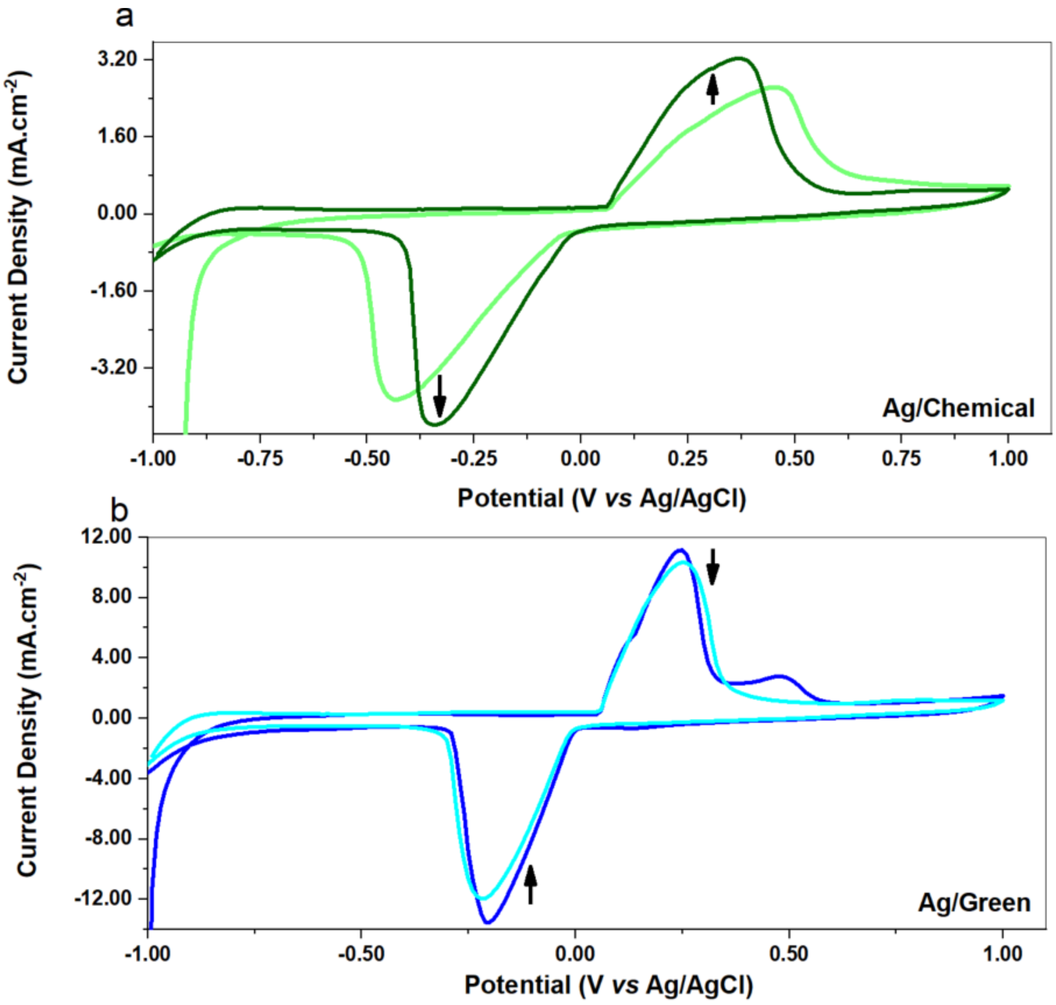

Figure S2. Stability of sensor structures after 25 cycles a) C-Ag b) G-Ag.(1 M KCl, 0.01 M BA and 50 mV/s)
